# Draft Genome Sequences of *Aggregatibacter actinomycetemcomitans* Strains 310a and 310b

**DOI:** 10.1128/genomeA.01282-17

**Published:** 2017-11-22

**Authors:** Anthony C. May, Hiroyuki Ohta, Hiroshi Maeda, Susumu Kokeguchi, Carla Cugini

**Affiliations:** a Department of Oral Biology, Rutgers School of Dental Medicine, Newark, New Jersey, USA; b United Graduate School of Agricultural Science, Tokyo University of Agriculture and Technology, Tokyo, Japan; c Ibaraki University College of Agriculture, Ibaraki, Japan; d Department of Endodontics, Osaka Dental University, Hirakata, Osaka, Japan; e Department of Oral Microbiology, Graduate School of Medicine, Dentistry and Pharmaceutical Sciences, Okayama University, Okayama, Japan

## Abstract

We report the draft genome sequences of *Aggregatibacter actinomycetemcomitans* strains 310a (310-TR) and 310b (310-OS). Strain 310a is a clinical isolate with a rough phenotype. Strain 310b is a laboratory-adapted isolate derived from the passage of 310a and displays a smooth phenotype.

## GENOME ANNOUNCEMENT

*Aggregatibacter actinomycetemcomitans* is a Gram-negative, nonmotile, facultative anaerobe of the oral microbiota implicated in the development of localized aggressive periodontitis (1, 2). *A. actinomycetemcomitans* strain 310a is a well-characterized clinical isolate that displays the classically observed star-shaped colony morphology (3). Examination by electron microscopy revealed that cells were highly fimbriated, which is a necessary factor for adherence and biofilm formation and characteristic of the translucent, “rough” phenotype (TR) (3, 4). Strain 310b is a laboratory-adapted isolate derived from 310a that has lost the classic *A. actinomycetemcomitans* phenotype due to multiple passages; strain 310b displays an opaque smooth (OS) colony morphology and is nonadherent (3). Although characterization of the rough-to-smooth transition has been explored in a number of studies, none have examined whole-genome sequences derived from rough and smooth isolates of a common origin (3–6). The sequences described here may allow for the identification of additional factors that contribute to the transition.

*A. actinomycetemcomitans* 310a and 310b were cultivated as previously described (3, 4). Briefly, these strains were grown in TSBY broth (3% tryptic soy broth [Difco Laboratories, Detroit, MI, USA] and 0.5% yeast extract [Difco] supplemented with 0.4% sodium bicarbonate) or on TSBY agar at 37°C in an anaerobic chamber (80% N_2_, 10% H_2_, and 10% CO_2_) (Model ANX-1, Hirasawa, Tokyo). Strain 310a cells (biofilm cells) were mechanically detached from glass tubes, sonicated, and collected by centrifugation. Strain 310b cells (planktonic cells) were collected by centrifugation. Genomic DNA was purified using the NucleoSpin tissue kit (Takara Bio, Inc., Japan) according to the manufacturer’s instructions.

Genomic libraries containing 150- to 550-bp inserts were constructed using the KAPA HyperPlus library kit. *A. actinomycetemcomitans* 310a and 310b genomes were paired end sequenced using the Illumina MiSeq platform. Read processing, base error correction, and *de novo* contig and scaffold assembly were automated with the A5-MiSeq pipeline (v20150522) (7, 8) (https://sourceforge.net/projects/ngopt/). QUAST v4.5 (9) was used to check the quality of the assembly and compare contig and scaffold statistics (http://quast.sourceforge.net/quast). Strain 310a contained 1,985,168 reads (*N*_50_ = 89,403 bp), producing 115 contigs and 64 scaffolds (longest scaffold = 226,154 bp) with 127-fold coverage for error-corrected bases. Strain 310b contained 1,816,022 reads (*N*_50_ = 95,018 bp) producing 112 contigs and 68 scaffolds (longest scaffold = 224,126 bp) with 111-fold coverage for error-corrected bases.

The final assembly resulted in 2,332,000 and 2,330,926 bp for 310a and 310b, respectively, with a GC content of 44.2%, consistent with NBCI-deposited *A. actinomycetemcomitans* genomes. To confirm taxonomic classification and identify lateral gene transfer events, all scaffolds were aligned to the nonredundant-microbial_20140513 reference database using the DC-MegaBLAST algorithm with the Taxator toolkit (v 1.3.3e) (10) (https://research.bifo.helmholtz-hzi.de//sotware; https://github.com/fungs/taxator-tk). All classified regions of both genomes belonged to the species *Aggregatibacter actinomycetemcomitans* (11.47% for 310a and 11.31% for 310b). Annotation was performed by NCBI using the Prokaryotic Genome Automated Annotation Pipeline (PGAAP) (best-placed reference protein, GeneMarkS+, v4.2) (11).

### Accession number(s).

The draft genome sequences of *A. actinomycetemcomitans* strains 310a and 310b have been deposited in GenBank under the accession numbers PCGV00000000 and PCGW00000000, respectively. The versions described here are the first versions.
